# Analysis of Moral Disengagement as a Modulating Factor in Adolescents’ Perception of Cyberbullying

**DOI:** 10.3389/fpsyg.2019.01222

**Published:** 2019-05-28

**Authors:** Isabel Cuadrado-Gordillo, Inmaculada Fernández-Antelo

**Affiliations:** Department of Psychology and Anthropology, University of Extremadura, Badajoz, Spain

**Keywords:** cyberbullying, adolescent, moral disengagement, moral identity, mediation effect

## Abstract

There have been various studies establishing a relationship between moral reasoning and the perpetration of cyberbullying, but very few analyzing either the moderating role played by moral disengagement in how both aggressor and victim perceive cyberbullying, or the repercussions of this moderation for the determination of the prevalence of the problem and for the design of prevention programs. The present study examines the relationship between moral disengagement, moral identity, and how victims of this type of abuse perceive cyberbullying. The participants were 1912 adolescents (51% women) from Extremadura (Spain) of ages from 14 to 18 years. They completed three questionnaires addressing perception of cyberbullying, moral disengagement, and moral identity. Factorial, structural, correlation, and hierarchical multiple regression analyses were used to construct their perceptual structure of cyberbullying. These analyses showed the influence of their different levels of moral disengagement on those perceptions, and the moderating role that moral identity plays in the direct and indirect relationships between moral disengagement and the perception of cyberbullying. They revealed, on the one hand, the key and the subsidiary criteria victims use to classify some given cybernetic behavior as a case of cyberbullying, and, on the other, that the victims’ levels of moral disengagement explain both the justifications they resort to in order to interpret occurrences of cyberbullying and their shifting or spreading of responsibility onto others. Finally, the results can be a key element in the design of effective psychological interventions aimed at improving adolescents’ moral identity in situations of cybernetic victimization.

## Introduction

In the last two decades, there has been an exponential growth in studies addressing the cyberbullying phenomenon, and an ever-greater diversity of variables introduced for analysis. Understanding why adolescents become aggressors or victims and what the factors are that favor the persistence of their roles are still difficult questions to answer. Once past the simple causal explanations, one has to opt for an interrelation of factors or components that offers a more holistic understanding, and allows better adjustment of cyberbullying prevention and intervention programs. The consideration of such variables as morality, prevalence, and perceptions about cyberbullying, addressing them all in an interrelated manner, is an as yet little explored area whose results could lead to advances in the understanding of the processes of aggression and victimization.

### Adolescents’ Perception of Cyberbullying

In recent years, there has been a proliferation of works addressing how adolescents perceive cyberbullying (Menesini et al., 2012; Dredge et al., 2014; Correa and López, 2018; Midamba and Moreno, 2019). Their results differ significantly due to the variety of instruments used, the samples selected, and the types of analysis applied. Nonetheless, they all coincide in pointing to knowledge of how young people define and identify the cyberbullying phenomenon and the different forms in which it manifests itself as constituting a powerful tool with which to adjust calculations of its prevalence, and to design specific measures of prevention and intervention in this type of abusive situation.

Researchers use a particular set of criteria to differentiate an episode of cyberbullying from an act of cyber-aggression – power imbalance, intentionality to hurt, repetition, publicity, and anonymity (Thomas et al., 2015). Adolescents have not only established a hierarchy of these criteria (Talwar et al., 2014; Barlett et al., 2016; Wright et al., 2017; Samoh et al., 2019), but have also constructed synergistic pairwise relationships among them (Nocentini et al., 2010; Palladino et al., 2017; Fernández-Antelo and Cuadrado-Gordillo, 2018). In this sense, studies indicate that although adolescents point to repetition of the aggression as being an identifying criterion for cyberbullying (Thomas et al., 2017), they generally consider it to be a second-order factor dependent on other primary factors such as publicity or intentionality to hurt. The results of Hutson (2016) reveal that adolescents tend to downplay the repetition of an aggressive behavior, arguing that when a single abuse goes viral in an uncontrolled way it can cause recurrent harm similar to that experienced when the aggression is suffered repeatedly. However, they perceive publicity as being a key element in the identification of cyberbullying. They understand that when abuses are committed in private, they can be classified as aggressions but not as cyberbullying because they do not cause the same pain as if the abuse transcends into the public plane through its diffusion with the use of technological resources (Chen and Cheng, 2016; Wright et al., 2017).

Likewise, adolescents also tend to establish a relationship between repetition and intentionality because they understand that when an aggressive behavior occurs continuously it can not be interpreted as unintentional (Menesini et al., 2012). However, some researchers warn of the difficulty that adolescents have in perceiving the intentionality of the aggressor in cybernetic contexts, as well as their tendency to justify or minimize the intentionality of these abusive acts by alluding to the manifestation of social interaction patterns among adolescents (Cuadrado-Gordillo and Fernández-Antelo, 2016). The adoption of maladaptive styles of humor or the normalization of aggressive behavior may explain the emergence of distorted interpretations of adolescent behavior (Sari, 2016; Betts and Spenser, 2017).

Another pair of criteria that adolescents associate together are anonymity and power imbalance, understanding that the lower a person’s skills in technological resources the less likely they will be able to uncover the authorship of cyber attacks (Palladino et al., 2017). Knowing that they can hide their identity, some adolescents perpetrate abuses that they would not dare to do in face-to-face contexts. Nevertheless, many victims have well-founded suspicions about the identity of their aggressors because they both generally belong to the same social or school circle.

Advances in the study of perceptions about cyberbullying have also revealed that the role which is played exerts a differential influence on which criteria are selected and prioritized (Dredge et al., 2014). While the victim emphasizes the intentionality to hurt, and associates it with the publicity of the abusive behavior, the aggressor stresses the imbalance and anonymity criteria (Compton et al., 2014; Crosslin and Golman, 2014; Fernández-Antelo and Cuadrado-Gordillo, 2018).

### Moral Variables and Cyberbullying: A Complex, as Yet Uncovered, Web

#### Moral Disengagement

Research studies directed at analyzing the explanatory causes of aggressive processes, whether offline or online (Pornari and Wood, 2010; Menesini et al., 2013; Gini et al., 2014; Leduc et al., 2018; Simao et al., 2018), have emphasized the moral variables involved. One of these variables is moral disengagement. This refers to the process by which individuals separate their personal moral norms from their immoral behaviors (Bandura et al., 1996). For decades, it has been known that moral disengagement is strongly related to bullying, and can even be a predictor of it (Ortega-Ruiz et al., 2002; Li and Lei, 2004; Gasser and Keller, 2009; Wang et al., 2017). In particular, the aggressor can, by activating certain mechanisms designed to release the tension caused by the contradictions that arise between their moral principles and their actions, intimidate others without feeling remorse (Hymel and Bonano, 2014; Allison and Bussey, 2017). These mechanisms correspond to four loci of behavior which allow an individual to regulate their conduct: justifying the behavior, shifting responsibility, minimizing the harm caused, and moving the causal focus onto the victim (Bandura et al., 1996). Despite the numerous studies that have addressed this topic, there has however, been very little work on whether these moral imbalances are also present in the figure of the victim, and whether they contribute to perpetuating the victim’s role (Hood and Duffy, 2018; Thornberg et al., 2018). The moral disengagement process in the victims would consist in their search for explanations that both justify they’re not confronting the aggressions they suffer and minimize their moral self-sanctioning. In this way, victims can disengage themselves morally so as to justify their inaction and even the aggressions they have suffered (Allison and Bussey, 2017; Luo and Bussey, 2019). Unlike the studies focused on the figure of the aggressor whose results tend to be mutually coincident, those that also include the victims have reported results and drawn conclusions that differ. Not only that, but most of the latter studies of this latter type deal with the dual role of victim and aggressor (Perren et al., 2012; Fanti and Kimonis, 2013), with works whose focus has been entirely on the figure of the victim being few and far between.

The study of moral disengagement has traditionally been linked to contexts and phenomena such as bullying that are face-to-face. However, the coexistence of both off- and on-line scenarios has led to the study of moral disengagement being transferred from physical contexts linked to bullying to cybernetic contexts associated with cyberbullying. Despite this, the influence that contextual factors may have on the interpretation of external signals has not yet been taken into account (Luo and Bussey, 2019). While some workers apply the same instruments for the assessment of the two types of abuse (Wang et al., 2016; Yang et al., 2018), others argue that they are distinct phenomena which require different approaches and instruments (Gini et al., 2014; Udris, 2014; Leduc et al., 2018; Meter and Bauman, 2018). In this sense, Harrison (2016) warns that the contextual factors which are so characteristic of online scenarios (e.g., the possibilities of anonymity, publicity, and the mass dissemination of messages or other types of audiovisual content) can contribute to certain abuses being committed that would not be committed in face-to-face situations. In parallel, these factors could contribute to the activation of moral disengagement mechanisms related to ignorance of the harm caused or to the diffusion of responsibility.

In spite of the initiatives being made to incorporate new approaches to the study of moral disengagement in response to the contextual duality in which we are immersed (offline and online), the aggressor continues to be given protagonism to the detriment of the study of the other roles involved in cyberbullying situations. While some recent research has opted to analyze the process of moral disengagement in adolescents who witness cyber abuse (DeSmet et al., 2016; Song and Oh, 2018; Luo and Bussey, 2019), the victims still seem to be forgotten, thus ignoring the possible repercussions that moral variables may have on the processes of victimization. Although timid, some approaches to the study of the association between cyber-victim and moral disengagement note that adolescents who are subjected to cyber abuses resort to a search for moral justifications, and develop a special empathy toward other victims so as to mitigate their self-attacks on their own self-esteem (Perren et al., 2012).

#### Moral Identity

In recent times, studies on moral disengagement and cyberbullying have incorporated a new variable into the web of relationships between morality and involvement in violent cyber behaviors: moral identity. Aquino and Reed (2002) define it as the process of self-regulation that motivates individuals to moral action, favoring a social identification that they use to construct their identity or self-definition (Hardy and Carlo, 2011). Likewise, it implies a personal commitment that generates high levels of well-being and protects the individual from others insofar as there is coherence between the behaviors manifested and the commitment that is taken on. In this sense, moral identity becomes the best predictor of moral action and commitment (Damon and Hart, 1992). Therefore it should be understood that persons who feel that moral values are key elements in defining their identity have a solid moral identity that favors their prosocial and positive interactions with others, and consequently a lower register of antisocial behavior (Hertz and Krettenauer, 2016). Determining the moral values which adolescents take on as being their own, which identify them to their peers, and which orient their behavior in a certain direction is a preliminary step to understanding the appearance of contradictions, inconsistencies, and distortions in their cognition and behavior. Aggressive and immoral actions can arise as a result of these dissonances. One of them is cyberbullying.

Studies that analyse the relationship between moral identity and the manifestation of violent and abusive behavior show that low levels of moral identity correlate positively with a high tendency toward antisocial actions (Hardy et al., 2014). When these violent behaviors are particularized in episodes of cyberbullying, and the effect of other moral variables such as moral disengagement are considered, researchers such as Hardy et al. (2015) or Aquino et al. (2007) note that moral identity can mitigate the influence of moral disengagement on cyberbullying. The emergence of new virtual scenarios has modified the way in which we relate, communicate, help, and also attack. To be able to understand the interpersonal, interactive dynamics emerging from this new context, it is necessary to continue deepening into the study of moral variables, particularly into the role of moral identity since, as Hertz and Krettenauer (2016) point out, the construction of solid moral identifiers can make it easier to access the structures and schemes of knowledge which guide the self-regulation of behavior and encourage moral action.

### The Study

The introduction of moral variables into studies of the prevalence of cyberbullying has provided important explanatory and causal indicators regarding the involvement of adolescents in cyber-aggression (Hardy et al., 2015; Allison and Bussey, 2017; Larranaga et al., 2018). These results have very limited applicability, however, unless other factors that exert a determining influence on the prevalence of aggression and victimization and on the persistence of these roles are taken into account at the same time. We refer to the perception that young people have of the different types of cyberbullying (Menesini et al., 2012; Dredge et al., 2014; Fernández-Antelo and Cuadrado-Gordillo, 2018). Recent studies have explored the relation between moral disengagement and perceptions of cyberbullying. They note that adolescents’ resort to various types of moral justification so as to interpret cyber abuse as jokes arising from the adoption of maladaptive styles of humor (Yang et al., 2018). However, there is still much ground to be explored to know how moral variables influence the self-regulation of the perceptive structure of cyberbullying through the selection, prioritization, and relation of the criteria identifying this phenomenon. A real challenge is to analyze the combination of moral variables and the perception of cyberbullying, and the synergy that arises between them. Without doubt, there will be important contributions made to allow an advance in the understanding of the processes of aggression and victimization. But an even greater challenge is to cede the protagonism to the victims, the forgotten agonists in studies which include moral variables. In this sense, the objectives of the present work were the following: (i) to identify the perceptive structure that cyberbullying victims have of this phenomenon, and that differentiates it from other forms of cyber aggression; (ii) to analyze the mediating effect of moral disengagement on the relationship between the perception of cyberbullying and victimization; and (iii) to explore the moderating role of moral identity in the relationship between the perception of cyberbullying and cybervictimization via moral disengagement. To respond to these objectives, we formatted the following hypotheses:

H1: The victims’ perception of cyberbullying will consist primarily of three factors: intentionality to hurt, imbalance, and publicity.
H2: Moral disengagement will exert a mediating effect on the relationship between the perception of cyberbullying and cybervictimization.
H3: Moral identity will moderate the relationships between the perception of cyberbullying and cybervictimization via moral disengagement.

## Materials and Methods

### Participants

The sample consisted of 1912 adolescents (51% boys and 49% girls), of ages from 14 to 18 years (*M* = 15.8; SD = 0.9). The sample selection followed an approximately proportional stratified procedure that included 21 lower and upper secondary schools in both urban and rural populations located throughout the Region of Extremadura (Spain). In the urban cases, the schools corresponded to both the center and the periphery of the town, so that the final overall sample would cover diverse socio-economic contexts with the participants’ families having highly varied academic levels. The inclusion of urban and rural areas had the objective of covering populations with very different family incomes. In the rural areas selected, the family income level was below the regional average, and approximately half of the participants’ parents had no university studies. In the urban areas, we selected schools located in residential areas, where there is a medium-to-high level of purchasing power, and schools located in humbler neighborhoods where people usually work in low-skilled jobs and where the family income level is medium-to-low. Despite these economic differences, all the participating adolescents had a smartphone. In total, 28 schools were selected and invited to participate in this study. This was done firstly through the Regional Educational Administration to which we had presented the research project and which facilitated access to the schools during school hours. And secondly, the researchers explained to the schools the objectives of the study and the use that would be made of the data, among other questions. This written invitation was followed by telephone and personal contacts so as to coordinate the collection of data. Seven of these secondary education schools declined the invitation to participate for various reasons, among which were the scarce availability of time especially for pupils in the higher years, the saturation of activities and surveys carried out during the school year, and the difficulties in coordinating the collection of data. For each school, one class was chosen at random from each of the 3rd year of Compulsory Secondary Education (ESO, lower secondary), the 4th year of ESO, the 1st year of Baccalaureate (upper secondary), and the 2nd year of Baccalaureate. According to the Regional Education Administration’s data, there were 23,842 adolescents enrolled in the aforementioned courses. We performed a representativity calculation by means of a statistical power analysis with a confidence level of 95% (α = 0.05), a power of 80% (β = 0.2), an effect of 0.120, and SD = 1. The result was a desired sample size of 2181 participants. In total, 2189 adolescents voluntarily completed the questionnaires that were distributed. The final sample after applying the data cleansing process was 1912 participants. The eliminated cases were those which were incomplete, which the pupil had used to joke with their peers by marking crosses in the form of some drawing, or which were improperly filled out in marking various responses where only one had been requested.

### Instruments

The instruments used for data acquisition were three questionnaires. The first was designed to identify cybervictims, and to determine their perceptions of cyberbullying on the basis of its defining criteria and the direct and indirect relationships that have been established between them (Fernández-Antelo and Cuadrado-Gordillo, 2018). A 4-value ordinal scale was used to calculate the prevalence of cybervictims: considering just the preceding 3 months, “never”, “once or twice”, “once a week”, and “several times a week.” An adolescent was considered to have been a victim of cyberbullying when they had been the object of one or more of the cyber-aggressions that were set out in the questionnaire at least “once or twice” in the preceding 3 months. In this study, we did not form any sample subgroups by frequency of the aggressions suffered. The review of the literature had shown that, in the cybernetic context, the criterion of repetition is sometimes displaced by that of publicity, and that adolescents can interpret and experience as cyberbullying episodes abusive behaviors that occur only once but quickly become viral. For this reason, we consider cybervictims to be those adolescents who have suffered “at least once” one or more of the aggressions presented. The inclusion in the questionnaire of a scale with different values provided information that is important for the adjustment of prevention and intervention programs, although these have not been analyzed in the present study. By way of example, the following is the questionnaire item that allowed us to identify the adolescents who consider themselves to be victims of cyberbullying. They were asked to indicate how often during the preceding 3 months they had suffered any of the following behaviors: “(1) I have been insulted through mobile phone or Internet; (2) I have been threatened or blackmailed through mobile phone or Internet; (3) lies and false rumors have been spread about me through mobile phone or Internet; (4) I have been removed from contact lists on social networks, group chats, or emails so as to exclude me; (5) I have had someone pretend to be me, and my email, private chat rooms, or social network profile have been accessed without my permission; (6) incriminating photos or videos, which are denigrating or demeaning to me, have been sent by mobile phone or Internet; (7) fights in which I participated have been recorded and spread through mobile phone, social networks, or other cyber means; (8) sexual or erotic type of content in which I took part has been sent out.”

The 25 items of this first questionnaire were grouped into eight thematic blocks corresponding to the different types of cyberbullying. A 5-value ordinal scale was used to indicate the degree of agreement with each item. The levels of internal consistency (Cronbach’s alpha) of each of the thematic blocks ranged from 0.69 to 0.81. An example of this type of item is: “Why do you think some peers threaten others through telephone calls? (1) Because they do not dare do it face to face for fear of reprisals; (2) Because they can hide their identity and inflict fear on others who are stronger; (3) Because it is the way they have of relating; (4) Because that way they feel more powerful; (5) Because it the only way they have to get what they want; (6) Because they feel more accepted by their friends; (7) Because it is a way of getting revenge; (8) Because they record the telephone calls and then spread them so that the victim repeatedly feels fear; (9) Because they like to see how others suffer; (10) They are jokes or other ways of having fun that are typical of adolescents.”

The second questionnaire was used to calculate the level of moral disengagement about cyberbullying. It was an adaptation of the questionnaire given in Bandura et al. (1996). We prepared it on the basis of other researchers’ adaptations of the original scale, adjusting the situation set out in each item to the cybernetic context. In particular, the adaptations of Bussey et al. (2015) and Meter and Bauman (2018) reduced the original scale to an 8-item questionnaire: moral justification, euphemistic language, advantageous comparison, displacement of responsibility, diffusion of responsibility, distortion of consequences, attribution of blame, and dehumanization of the victim. The adaptation made by Day and Lazuras (2016) consisted of 15 items, but with the analysis of disengagement mechanisms being reduced to just 4: minimization of harmful effects, moral justification, denial of responsibility, and dehumanization. Following the same procedure as in the aforementioned works, we chose to adapt the 32 items of the original scale by replacing aggressions linked to the off-line context with other on-line ones. The items from Meter and Bauman (2018) and Day and Lazuras (2016) were included without change (e.g., “Cyberbullying should be justified if you have been mistreated by others”, “Some people can’t be hurt by cyberbullying because they lack feelings”, “Cyberbullying annoying classmates is just teaching them a lesson”, “If people give out their passwords to others, they deserve to be cyberbullied”). And we made our own specific adaptations of the rest (e.g., “Sending humiliating photos or re-tweeting false messages about someone is just a form of fun or joking”), until completing the 32 of the original scale. The coefficient of reliability (Cronbach’s alpha) was α = 0.84. A 5-value ordinal scale was used to indicate the degree of agreement with each item, ranging from “strongly disagree” to “strongly agree.” Before its definitive application, the questionnaire was subjected to a confirmatory factor analysis with part of the sample (*n* = 325) to verify the existence of the eight factors (disengagement mechanisms) and their associated items. The objective of this first analysis was to ensure the validity of the questionnaire before it was distributed. The results showed the fit to be adequate: χ^2^(17, *N* = 325) = 138.61, *p* < 0.001, CFI = 0.97, TLI = 0.98, RMSEA = 0.05. It was therefore decided that the questionnaire was appropriate for use in the study. Once all the questionnaires had been collected and entered into the database, a new confirmatory factor analysis was carried out to ensure for the second time the validity of this instrument and the permanence of the eight initial factors. The level of fit obtained was satisfactory: χ^2^*/*df = 1.977, *p* < 0.001, CFI = 0.95, TLI = 0.97, RMSEA = 0.043.

The third questionnaire was that of Aquino and Reed (2002) designed to measure the level of moral identity. The participants had to express their degree of agreement with 10 items forming the questionnaire, again using a 5-value ordinal scale ranging from “strongly disagree” to “strongly agree.” Each item included the term “these characteristics” (e.g., “It would make me feel good to be a person who has these characteristics”). The participants were asked to replace this term with the following list of adjectives: caring, compassionate, fair, friendly, generous, helpful, hard-working, honest, and kind. Their responses reflected the “self-importance” that they attribute to these characteristics in terms of moral identity. This questionnaire has two dimensions: internalization and symbolization. The first, internalization, addresses the degree to which moral traits are fundamental for the self-concept, thus constituting the aspect of moral identity that is most private. The second, symbolization, represents the degree to which the traits are reflected in an individual’s actions, through which others are going to identify him or her and attribute certain characteristics to that person. Therefore, this dimension reflects the more social aspect of moral identity. The coefficient of reliability of the scale was α = 0.81. In order to calculate the goodness of fit of the two-factor model, a confirmatory factorial analysis was performed with the entire sample, obtaining a satisfactory fit: χ^2^*/*df = 1.489, *p* < 0.001, CFI = 0.953, TLI = 0.957, RMSEA = 0.041.

### Procedure

Prior to the distribution of the questionnaires to the adolescents, both the research objectives and the procedure, instruments and techniques used were checked and approved by the Bioethics and Biosafety Committee of University of Extremadura (Spain). Also, the parents’ approval was required (as the study was dealing with minors) as also was that of the Regional Education Administration (from both the school inspectors and the schools’ headteachers). In the case of the parents, they were sent a letter describing the nature of the investigation and the mechanisms used to guarantee the anonymity and confidentiality of their children’s responses. Specifically, they were informed that their children would not have to write their names or other identifying information about their family. They were also informed that the distribution, collection, storage, and analysis of the responses would be carried out by the research team responsible for the project, and that no teacher or other person from the school would read the responses their children gave to the questionnaires. This letter was accompanied by a written informed parental consent that they were to send back to the school if they wanted their child to be part of the study sample. In the case of the Education Administration, obtaining approval consisted of two phases. In the first, a detailed report of the objectives and methods of the investigation was sent to the Inspection Service of the Regional Government, together with the ethical principles conforming it. Approval of this report allowed access to the Region’s schools for distribution of the questionnaires. The second phase required acceptance on the part of the selected schools’ directive teams to facilitate the choice of classrooms and access to them during school hours.

Once all the favorable permissions had been obtained, the questionnaires were handed out by the researchers who remained in the classrooms while the adolescents completed them, and then gathered the completed questionnaires in. In this way, the confidentiality of the data was guaranteed, and any doubts the respondents had about any term or wording in the items could be answered.

The participants had 50 min to answer the questionnaires, although the average time spent was around 20 min. The data collection process, once all the permissions and authorizations were obtained, lasted for 4 months (February to May 2018), adapting to the times and schedules that the schools themselves indicated.

### Data Analysis

Accessing the victims’ perceptive structure about the cyberbullying phenomenon required the construction of a structural model based on a confirmatory factorial analysis. The structural equation model resulting from this analysis was subjected to maximum likelihood estimation. To check the fit, we used the χ^2^ statistic, the comparative fit index (CFI), the goodness-of-fit index (GFI), the Tucker-Lewis index (TLI), the root mean square error of approximation (RMSEA), and the root mean square residual (RMR). We also estimated the model’s standardized regression coefficients. To analyze the mediation effect of moral disengagement in the relationship between the perception of cyberbullying and cybervictimization, we applied the mediation test of Baron and Kenny (1986). This test requires there to be significant relationships between perception and cybervictimization, perception and moral disengagement, and moral disengagement and cyberbullying while controlling for the perception variable. Likewise, it requires there to be a significant coefficient of the indirect effects between perception and cybervictimization via moral disengagement, a condition whose satisfaction was verified by the bias-corrected percentile bootstrap method. Finally, to determine whether this mediation process was moderated by the moral identity variable, we resorted to the moderated mediation test of Hayes (2013).

## Results

### Perception of Cyberbullying: Explanatory Model

The results revealed the existence of 316 adolescents who claimed to have suffered one or more cyber-attacks at least once or twice in the preceding 3 months. It is important to point out that this group of victims did not include those adolescents who claimed to be both victims and aggressors.

**FIGURE 1:**
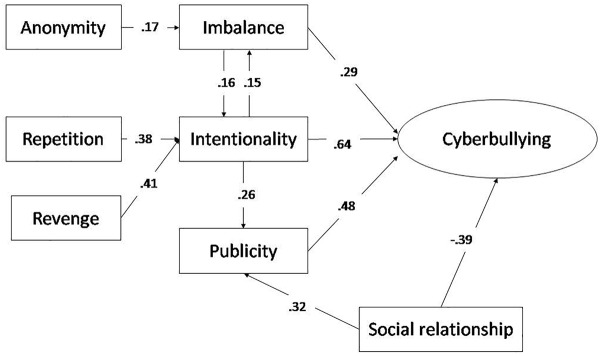
Structural equation model of the cyberbullying victim.

The confirmatory factorial analysis of the dimensions that form the perceptive structure of the victims on cyberbullying (χ^2^/df = 1.654, *p* < 0.01, RMSEA = 0.039, RMR = 0.027, CFI = 0.952, TLI = 0.948, GFI = 0.946) together with the correlation analysis of the variables constituting those dimensions (Table 1) allowed a structural model to be constructed which comprised seven standardized observable variables and one latent variable, cyberbullying (Figure 1). The fitting indices calculated showed the fit of the model to be correct: χ^2^ = 24.579; χ^2^/df = 1.928, *p* < 0.05; RMSEA = 0.042; RMR = 0.009; CFI = 0.965; TLI = 0.974; GFI = 0.970; NFI = 0.968. To verify that the resulting model was not over-fitted, the parsimony-adjusted indices were calculated: PGFI = 0.59; PNFI = 0.68.

**Table 1:** Correlations between the variables that form the victims’ perception of cyberbullying behavior.

	1	2	3	4	5	6	7	8
(1) Imbalance	–							
(2) Intentionality	0.51^**^	–						
(3) Repetition	0.18	0.41^**^	–					
(4) Publicity	0.32^*^	0.68^***^	0.64^***^	–				
(5) Anonymity	0.63^***^	0.39^**^	-0.28^*^	-0.22	–			
(6) Revenge	0.38^**^	0.32^*^	0.16	0.09	0.02	–		
(7) Social relationship	-0.29^*^	-0.61^***^	0.42^**^	0.40^**^	0.14	-0.38^**^	–	
(8) Cyberbullying	0.33^*^	0.72^***^	0.15	0.16	0.10	0.19	-0.48^**^	–

*^∗^p < 0.01; ^∗∗^p < 0.01; ^∗∗∗^p < 0.001.*

The standardized regression coefficients reflected a network of predictors of cyberbullying consisting of intentionality (β = 0.638, *p* < 0.001), imbalance (β = 0.289, *p* < 0.05), and publicity (β = 0.481, *p* < 0.01), and an inverse relationship between the variable “social relationship” and cyberbullying (β = -0.387, *p* < 0.01). Other results showed a web of indirect influences that reflect the complexity of the model guiding adolescents’ perception of cyberbullying (Figure 1). For instance, there were strong associations of the variables “repetition” (β = 0.383, *p* < 0.05) and “revenge” (β = 0.407, *p* < 0.01) with the variable “intentionality.” There was also an influence of “anonymity” on “cyberbullying” through the variable “imbalance” (β = 0.168, *p* < 0.05). The results clearly indicate interactions among the main predictors of cyberbullying (Figure 1).

The cybervictims’ perceptive structure reflects the existence of three fundamental criteria: intentionality, publicity, and imbalance. These explain 48% of the variance of the cyberbullying variable. In this study therefore, the confluence of these three criteria will constitute a new variable that we shall denote “perception of cyberbullying.”

### Mediation Effect of Moral Disengagement in the Perception of Cyberbullying

To detect the mediation effect that the variable “moral disengagement” may have in the relationship between the perception of cyberbullying and cybervictimization, we applied the four-step mediation test of Baron and Kenny (1986), with regression analyses performed in each of the steps. The first step (Model 1) showed a strong positive association between the perception of cyberbullying and cybervictimization (Table 2). The second step (Model 2) showed perception to be negatively associated with moral disengagement (β = -0.47, *p* < 0.001). The regression coefficients resulting from the third step (Model 3) showed there to be an association between moral disengagement and cybervictimization (Table 2). In the fourth step, it is was verified that, controlling for the “perception” variable, the effect of moral disengagement on cybervictimization remained significant, evidence for its mediatory action. Finally, we calculated the indirect effects so as to avoid Type II errors. For this purpose, we applied the percentile bootstrap method. The results indicated that the indirect effect of perception on cybervictimization via moral disengagement was significant (β = 0.19, SE = 0.03, 95% CI = [0.06, 0.27]). The mediation effect represented 43.16% of the total effect, thus confirming its satisfactoriness.

**Table 2:** Mediation effect of perception on cyberbullying.

Predictors	Model 1 (cyberbullying)		Model 2 (moral disengagement)		Model 3 (cyberbullying)	
	β	*t*	β	*t*	β	*t*
Perception	0.39	8.54^∗∗∗^	-0.47	-11.03^∗∗∗^	0.31	5.94^∗∗∗^
Moral disengagement					0.38	7.14^∗∗∗^
*R*^2^	0.22		0.29		0.35	
*F*	38.19^∗∗∗^		49.07^∗∗∗^		44.56^∗∗∗^	

*^∗∗∗^ p < 0.001. Each column is a regression model that predicts the criterion at the top of the column.*

### The Role of Moral Identity in the Relationship Between the Perception of Cyberbullying and Cybervictimization

**FIGURE 2:**
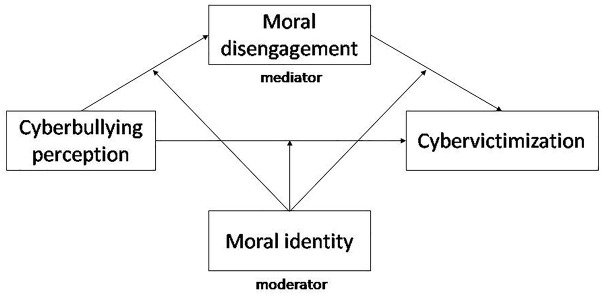
Moderated mediation model.

Starting from the verified model of direct and indirect relationships between the perception of cyberbullying and cybervictimization through the mediation of the variable “moral disengagement”, we analyzed the moderating influence that moral identity might have in this web of relationships. Following the procedure put forward by Hayes (2013), we established three regression models with which to analyze the moderator effects of moral identity: Model 1, for the relationship between perception and cybervictimization; Model 2, for the relationship between perception and moral disengagement; and Model 3, for the relationship between moral disengagement and cybervictimization (Figure 2).

The results (Table 3) indicated that perception has a significant influence on cybervictimization (β = 0.47, *p* < 0.001) moderated by moral identity (β = 0.30, *p* < 0.001; Model 1). Simple slopes were calculated for one standard deviation both above and below the mean. In particular, the results showed that low levels of moral identity lead to a more poorly defined identification of cyberbullying criteria, and that this is associated with lower levels of cybervictimization (β_simple_ = 0.48, *p* < 0.01). Similarly, high levels of moral identity imply a sharper definition of the perception of cyberbullying that is associated with higher levels of cybervictimization (β_simple_ = 0.61, *p* < 0.001).

**Table 3:** Moderated mediation effect of perception on cyberbullying.

Predictors	Model 1 (cyberbullying)		Model 2 (moral disengagement)		Model 3 (cyberbullying)	
	β	*t*	β	*t*	β	*t*
Perception	0.47	7.36^***^	-0.32	-5.86^***^	0.31	5.94^***^
Moral identity	-0.58	9.84^***^	-0.35	-6.04^***^	-0.41	-7.21^***^
Perception × Moral identity	0.30	5.63^***^	0.21	3.74^**^	0.27	4.63^***^
Moral disengagement					0.39	6.58^***^
Moral disengagement × Moral Identity					-0.11	1.44
*R*^2^	0.39		0.42		0.36	
*F*	52.84^***^		59.14^***^		48.07^***^	

*^∗∗^p < 0.01; ^∗∗∗^p < 0.001. Each column is a regression model that predicts the criterion at the top of the column.*

Model 2 reflects a significant influence of the perception of cyberbullying on moral disengagement (β = -0.32, *p* < 0.001), with moral identity exerting a moderating effect (β = 0.21, *p* < 0.01). The results indicate that low levels of moral identity imply a more poorly defined perception of cyberbullying associated with higher levels of moral disengagement (β_simple_ = 0.25, *p* < 0.05). Similarly, higher levels of moral identity confirm a significant effect of perception on moral disengagement (β_simple_ = 0.20, *p* < 0.05).

Model 3 reflects an association between moral disengagement and cyberbullying (β = 0.39, *p* < 0.001), with no significant moderating effect of moral identity (β = -0.11, *p* > 0.05).

The indirect effects of the perception of cyberbullying on cybervictimization through moral disengagement and moderated by moral identity were calculated using the percentile bootstrap method. The results indicated that when there are low levels of moral identity then one finds a significant indirect effect of perception on cybervictimization through moral disengagement (β = 0.18, SE = 0.07, 95% CI = [0.03, 0.29]). There were also significant indirect effects when the levels of moral identity were high (β = 0.16, SE = 0.05, 95% CI = [0.08, 0.20]).

## Discussion

The social, economic, school, and health problems generated by cyberbullying have traversed all types of barriers and limits despite the countless attempts to combat them. This study has aimed at shedding some light on the understanding of victimization processes and the causes that motivate their persistence. The complex network of relationships that form the perceptive structure that victims have of cyberbullying reveals which are the first- and second-order criteria that they take as identifying and defining the phenomenon. Added to this complicated web of interactions is the mediating and moderating effect that some moral variables, such as moral disengagement and moral identity, can exert on the persistence of the role of victim.

### Factors That Articulate the Victims’ Perceptive Structure of Cyberbullying

The studies that address the conceptualization that adolescents make of cyberbullying indicate that anonymity is one of their main defining criteria of cyberbullying (Udris, 2014; Barlett et al., 2016; Samoh et al., 2019). Nevertheless, for Spanish adolescents who are victims of cyberbullying, the intentionality to cause harm is the key element that allows them to identify the presence of this phenomenon. This criterion is in turn reinforced by others such as repetition and revenge, i.e., a succession of aggressions suffered, or the perception of revenge in the aggressor as reflecting the existence of an express will to hurt. The formation of local social networks comprising persons they know well and with whom they maintain some kind of relationship in offline contexts may mean that these young people intuit the identity of their aggressors, and therefore relegate the anonymity criterion to a secondary level, which itself would be dependent on the degree of dominance that the aggressors have of the technologies involved to be able to hide their identity.

Publicity and imbalance of power constitute the principal axes of these adolescents’ perceptive structure about cyberbullying, and both are closely related to intentionality. These results are in line with those of previous work (Nocentini et al., 2010; Fernández-Antelo and Cuadrado-Gordillo, 2018) that, in a cybernetic context, those who want to hurt others must have sufficient technical knowledge to impersonate identities, manipulate images or videos, or eliminate other people from distribution lists and contacts, for example, and that it is the diffusion of these aggressions which demonstrates this intentionality at the same time as reinforcing it. In sum, these results allow us to determine that there are principally three factors which articulate the perception victims have of cyberbullying: intentionality to hurt, imbalance of power, and publicity. Hypothesis H1 is thus confirmed. There appears a secondary factor in this perceptive structure which we have denoted “social relationship”, representing the adolescents’ interpretation of certain aggressions as innocuous formulas of interaction or jokes. According to Betts and Spenser (2017) and Sari (2016), the adoption of maladaptive styles of humor or distorted perceptions about the abuses suffered can lead to the normalization of this type of behavior as typical patterns of adolescents’ socialization. The justification of the aggressions suffered through the activation of mechanisms of moral disengagement such as euphemistic language, for instance, reinforces the situation of victimization, especially its persistence over time (Chen et al., 2017; Yang et al., 2018). Precisely, it is moral disengagement or the absence of moral referents that explains why young people can classify the same behavior sometimes as a form of relating socially and other times as cyber-aggression.

### Mediating Effect of Moral Disengagement in the Relation Between the Perception of Cyberbullying and Cybervictimization

The present results confirm a significant relationship between moral disengagement and cybervictimization, reflecting that, as the level of moral disengagement increases, so does the prevalence of victims. These relationships had already been noted by Meter and Bauman (2018) and Yang et al. (2018) in their analyses of the influence of moral variables on the perpetration of cyber-aggression by adolescents. However, there have been very few preceding studies that put the protagonism on the victims, making it difficult to compare results.

Beyond the correlation study of these variables, the results show the mediating power exerted by moral disengagement in the relationship between the perception of cyberbullying and cybervictimization. Specifically, one can conclude that the type of perception that victims have of cyberbullying can facilitate the activation of certain mechanisms of moral disengagement (Model 1) such as, for example, euphemistic language, the distortion of consequences, or advantageous comparison. According to the social cognitive theory of Bandura (2002), selective recourse to these mechanisms allows victims to reduce the tension experienced when others do not respect their moral standards and they either feel unable to put a stop to the situation or do not dare to because they fear feeling excluded or making matters worse. In this way, the victims try to play down, camouflage, or distort the intentions behind the abuses they suffer, or the motivations that led the aggressors to disseminate these abuses by technological means. The apparent ignorance of the identity of the aggressor and the lack of direct contact between aggressor and victim (characteristics specific to cyberbullying) can foster this type of moral justification to escape the emotional self-sanctions imposed by not respecting their own moral standards (Perren and Gutzwiller-Helfenfinger, 2012).

The negative association between the perception of cyberbullying and moral disengagement (Model 2) shows that, as the victims more strongly identify the phenomenon with intentional aggressive episodes in which there is an imbalance of power in favor of the aggressor who resorts to dissemination of the abuses committed in order to increase the hurt done to the victim (Figure 1), there is less need to seek mechanisms of moral disengagement to justify the aggressor’s intentions, or a lessening of the consequences suffered when compared with others that some of their peers may be suffering.

The present results confirm that moral disengagement has a positive relationship with cybervictimization (Model 3), in the same way as other studies which have verified the existence of a relationship between moral disengagement and cyberbullying (Kowalski et al., 2014; Bussey et al., 2015; Meter and Bauman, 2018). This association indicates that the cognitive resources which the victims use to make the aggressions they experience seem less harmful, or not as harmful as other forms of abuse or delinquency, affect the indices of the prevalence of cybervictimization and the persistence of the role of victim. In trying to downplay the hurt suffered and to mask the processes of victimization to which they are being subjected, they significantly weaken their establishment of support networks. If a person hides or does not recognize their pain, they apparently do not need help from others to combat situations of helplessness, risk, or danger. In short, there is confirmation of the mediatory effect of the variable “moral disengagement” in the relationship between cybervictimization and the perception of cyberbullying, thus confirming hypothesis H2. The perception of cyberbullying becomes a predictor of cybervictimization by way of moral disengagement. Acting on these cognitive and moral distortions should be part of cyberbullying prevention and intervention programs so as to ensure their minimal efficacy.

### Moderating Effect of the Variable Moral Identity

Previous studies have pointed to the moderating influence that moral identity can have between certain personal variables and cyberbullying (Wang et al., 2017; Yang et al., 2018). The intention with this study was to define the complex web of relationships between moral identity, the perception of cyberbullying, and cybervictimization, without forgetting the mediating influence exerted by moral disengagement. In this sense, the results reveal the power of moral identity to moderate the relationship between perception and cybervictimization. High levels of moral identity strengthen a definition of cyberbullying based on the three key identifying criteria constituting its perceptive structure: intentionality, publicity, and imbalance. Likewise, this conceptual and perceptual delimitation, based on the values that form the backbone of the moral identity of cybervictims, results in their increased prevalence (Model 1). To the extent that they have solid criteria available to let them distinguish an aggression from an episode of cyberbullying, without needing to seek justifications that hide their helplessness or threaten their self-esteem, it would be simpler to identify them as victims, and the prevalence data would thus pick up cases that theretofore had remained hidden. A strong moral identity may thus improve access to the structures of knowledge and schemes that guide self-regulation, foster social action, and help define the situations of cyberbullying that these adolescents are suffering (Aquino and Reed, 2002).

The moderating effect of moral identity is also present in the relationship between the perception of cyberbullying and moral disengagement (Model 2). In this case, moral identity helps neutralize the negative effects of moral disengagement on the perception of cyberbullying. The results indicate that high levels of moral identity favor an adjusted perception of cyberbullying and lower levels of moral disengagement. These results are consistent with those of other studies (Hardy et al., 2015; Hertz and Krettenauer, 2016) that have explained how a well defined moral identity, based on values that favor pro-social interactions and combat violent behavior whether committed by or committed against the person, is negatively associated with the manifestation of mechanisms of moral disengagement. This capacity to neutralize the negative effect of moral disengagement reflects the self-regulatory capacity that moral identity can acquire to compensate for maladaptive social cognitions (Hardy et al., 2015).

**FIGURE 3:**
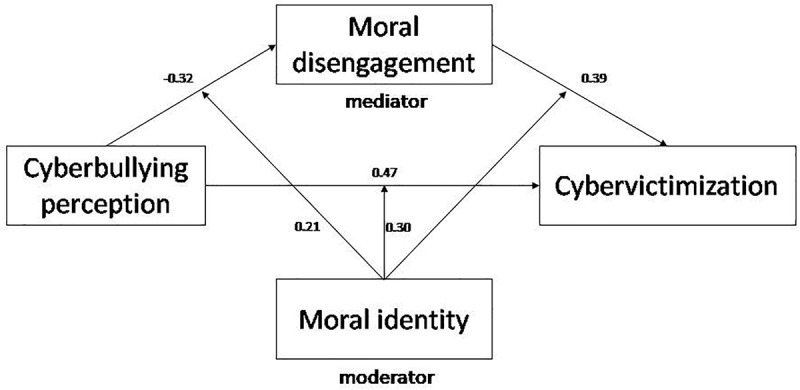
Moderated mediation model.

These moderating effects of moral identity do not appear in the relationship between moral disengagement and cybervictimization, where it was expected that its neutralizing role would be played with greater force (Figure 3). This means that hypothesis H3 is only partially confirmed. A possible explanation for this may lie in the distortions that the adolescents manifested in their interpretation of cyberbullying when they consider it to sometimes be harmless behavior typical of social relationships, jokes, or the adoption of maladaptive styles of humor.

Finally, the significance of the indirect effects of the perception of cyberbullying on cybervictimization via moral disengagement moderated by moral identity not only confirms that this last variable plays a moderating role, but also that it has a major predictive value.

## Conclusion

The association between moral variables and cyberbullying has been an object of study during the last decade. The role analyzed, however, has almost exclusively been that of the aggressor. One of the main contributions of this present study lies in the transfer of protagonism to the victim in an attempt to understand some of the causes that contribute to the lasting nature of their role. Furthermore, the adaptation of the scale of Bandura et al. (1996) to the study of moral disengagement in cybernetic contexts opens up new possibilities for analysis of the moral mechanisms that the different agents involved in cyberbullying episodes use to justify the facts or to dilute their responsibility, among other actions. Likewise, the consideration of more than one moral variable reflects the importance given to this dimension, and the attempt to seek more complex explanations removed from the establishment of simple, unidirectional relationships.

But undoubtedly the main contribution of this study has been to describe how moral identity moderates the association between the perception of cyberbullying and cybervictimization, taking into account the mediating power of moral disengagement.

### Limitations and Future Research

This work has some limitations. First, it was a cross-sectional study, so that there has to be caution in making any generalization of the results or in determining any causal and predictive relationships. And second, the analyses did not take age into account as a variable. Although the ages of the participants cover an interval that is not very broad (14–18 years), the evolutionary moment at which these adolescents find themselves may have had some sort of influence on the results. One has to assume that at the end of adolescence moral development is more settled than in mid adolescence, and this may affect the construction of identity and the use of mechanisms of moral disengagement.

These limitations serve to orient the consideration of new lines of research to gain deeper knowledge of the processes of cybernetic victimization. The adoption of a longitudinal approach covering the different evolutionary moments of adolescence and youth, and the inclusion of gender as a variable, would complete the results that have been presented here.

## Data Availability

The datasets generated for this study are available on request to the corresponding author.

## Ethics Statement

This study was carried out in accordance with the recommendations of “Bioethics and biosafety committee of University of Extremadura” with written informed consent from all subjects. All subjects gave written informed consent in accordance with the Declaration of Helsinki. The protocol was approved by the “Bioethics and biosafety committee of University of Extremadura”.

## Author Contributions

IC-G and IF-A designed the work, acquired and interpreted the data, and wrote and revised the manuscript.

## Conflict of Interest Statement

The authors declare that the research was conducted in the absence of any commercial or financial relationships that could be construed as a potential conflict of interest.
